# Implementation of an ePRO-based system supporting treatment readiness assessments in lung cancer care: a mixed-methods study

**DOI:** 10.1007/s11136-026-04363-0

**Published:** 2026-08-12

**Authors:** Lasse Lybecker Scheel-Hincke, Johanne Dam Lyhne, Sanne Skovbjerg, Anne Skov Villadsen, Rasmus Blechingberg Friis, Mette Bøg Horup, Anne Mette Ølholm

**Affiliations:** 1 CIMT - Centre for Innovative Medical Technology, Odense University Hospital, J.B. Winsløws Vej 4, Entrance 216, DK, 5000 Odense, Denmark; 2 Department of Clinical Research, University of Southern Denmark, Odense, Denmark; 3 Department of Oncology, University Hospital of Southern Denmark - Vejle, Vejle, Denmark; 4 Quality and Patient Safety, University Hospital of Southern Denmark - Vejle, Vejle, Denmark; 5 Department of Oncology, Aarhus University Hospital, Aarhus, Denmark

**Keywords:** Electronic patient-reported outcomes, Lung cancer, Implementation, Treatment readiness, Mixed-methods

## Abstract

**Purpose:**

Electronic patient-reported outcomes (ePROs) have shown clinical benefits in oncology. Although widely used for symptom monitoring during and after cancer treatment, their potential to replace existing workflows, such as treatment readiness assessment without a direct clinical encounter, remains underexplored. Real-world evidence on the feasibility, acceptability, and resource implications of such approaches is limited. This study evaluated the implementation and use of an ePRO-based system to support treatment readiness assessments in lung cancer care.

**Methods:**

A mixed-methods study guided by the RE-AIM framework was conducted in routine lung cancer care at a Danish oncology department. Quantitative data from the ePRO platform and electronic health records were used to assess uptake and questionnaire completion. Qualitative data from field observations and semi-structured patient interviews were used to examine workflow integration, user experiences, and perceived value. Changes in staff time use before and after implementation were estimated by combining registry data, field observations, and staff input.

**Results:**

A total of 704 patients completed at least one ePRO questionnaire between December 2020 and March 2024, corresponding to 6,134 completed questionnaires. Treatment-level completion increased from 17.7% to 76.3%. In practice, ePRO data were used alongside blood test results, prior medical history, and clinical judgement to assess treatment readiness. The ePRO-based workflow was estimated to reduce nurses’ time use by approximately 6 min per treatment. Patients and staff generally found the system meaningful and acceptable.

**Conclusion:**

The ePRO-based system appeared feasible to integrate into routine lung cancer care and supported more targeted treatment readiness assessments.

**Supplementary Information:**

The online version contains supplementary material available at https://doi.org/10.1007/s11136-026-04363-0.

## Abstract (plain language summary)

Patients receiving lung cancer treatment are often contacted by telephone before each treatment session to discuss symptoms and determine whether treatment can proceed as planned. This study evaluated a digital questionnaire system that allowed patients to report symptoms online before treatment, replacing the routine phone calls. The findings showed that the system could be integrated into routine clinical practice and helped healthcare staff focus follow-up contact on patients who needed additional assessment. Most patients and staff viewed the system positively and found it meaningful to use. The system also appeared to reduce the time nurses spent preparing patients for treatments. Some patients experienced difficulties using digital tools, highlighting the need to maintain non-digital alternatives. Overall, the study suggests that digital symptom reporting may help support and streamline cancer care when combined with clinical judgement and existing care routines.

**Supplementary Information:**

The online version contains supplementary material available at https://doi.org/10.1007/s11136-026-04363-0.

## Introduction

Electronic patient-reported outcomes (ePROs) enable patients to systematically report symptoms, functioning, and well-being directly to healthcare professionals, without interpretation by clinicians or others [1, 2]. In oncology, ePRO-based symptom monitoring has been associated with substantial clinical benefits, including improved symptom control [3], reduced emergency visits and hospital admissions [4]. Despite diverging results, some ePRO interventions have been shown to even improve survival [5–7]. Furthermore, systematic symptom reporting may improve the detection of under-recognised physical and psychological symptom burdens [8, 9] and strengthen communication between patients and clinicians [10].

Despite growing evidence of ePRO benefits [9], integrating ePRO systems into routine oncology practice remains challenging [11]. While feasibility has been demonstrated [12], consistent barriers persist: Incomplete integration with electronic health records, lack of alignment with existing clinical workflows, limited staff engagement, and concerns about added workload or patients’ digital literacy [13–15]. Furthermore, evidence from routine practice beyond pilot or trial settings remains sparse, especially regarding workflow integration and time use [16].

A promising and still under-examined application of ePRO systems is their potential to support or replace existing clinical workflows, such as assessing treatment readiness prior to systemic therapy. In routine oncology practice, this assessment is often performed through pre-treatment telephone calls or outpatient consultations. This process can be labour-intensive for staff and burdensome for patients, who may spend considerable time waiting for a call or travelling to the hospital. If ePRO systems, in combination with blood test results and prior clinical history, could provide clinicians with sufficient information to support treatment readiness assessments, this may help reduce clinical burden and improve efficiency without compromising quality of care.

To address this gap, the aim of this study was to evaluate the implementation and use of an ePRO-based system to support treatment readiness assessments in lung cancer care. By evaluating the routine use of this system in clinical practice, the study provides real-world evidence on how patient-reported data can be integrated into clinical workflows and potentially replace existing pre-treatment procedures in oncology care.

## Methods

### Study design and analytical framework

This study employed a mixed-methods design combining data from the ePRO platform, routinely collected electronic health records, structured observational fieldwork in the outpatient clinic, and semi‑structured patient interviews. The study was guided by the RE-AIM framework (Reach, Effectiveness, Adoption, Implementation, and Maintenance) [17]. The framework provides a structured approach for evaluating complex healthcare interventions in real-world settings and was used to guide the evaluation of the ePRO system in routine oncology practice. In line with the study aim, the evaluation focused on three RE-AIM dimensions considered most relevant for the present implementation: (1) Reach and adoption, assessing both patient-level uptake of the ePRO system and treatment-level completion of questionnaires prior to treatment sessions; (2) Implementation, describing integration into clinical workflows and implications for staff time use; and (3) Effectiveness (hereafter referred to as perceived effectiveness, as this study evaluated user experiences rather than clinical outcomes), exploring perceived value and impact among patients and staff.

### Setting and intervention

The study took place at University Hospital Southern Denmark - Vejle, a regional hospital and part of the University Hospital of Southern Denmark research collaboration. In December 2020, the Oncology Department implemented an ePRO system for patients receiving systemic therapy for lung cancer. The system was designed to replace routine pre-treatment telephone consultations by collecting structured symptom data directly from patients through an electronic questionnaire, thereby supporting clinical decision-making and patient-clinician communication.

Prior to implementation, all patients were contacted by telephone before each treatment session to assess symptoms, side effects, and general readiness. Information from these calls, together with blood test results and prior clinical history, guided decisions on whether treatment could proceed.

With the ePRO system, patients are asked to complete a 42-item questionnaire covering common side effects and clinical parameters two to three days before treatment, using the digital platform “Mit Sygehus” (My Hospital), accessible via mobile app or web. The questionnaire was developed based on a pre-treatment reporting instrument for cancer patients by AmbuFlex [18], a PRO competence centre in the Central Denmark Region, and was subsequently adapted for patients with lung cancer. The instrument combines selected items from the European Organisation for Research and Treatment of Cancer (EORTC) QLQ‑C30 [19], the EORTC QLQ‑CIPN20 [20], and the Eastern Cooperative Oncology Group (ECOG) Performance Status Scale [21]. In addition, the questionnaire includes target group-specific items developed by clinicians to ensure clinical relevance and comprehensive coverage of key symptoms and treatment-related adverse effects in patients with lung cancer.

**Fig. 1:**
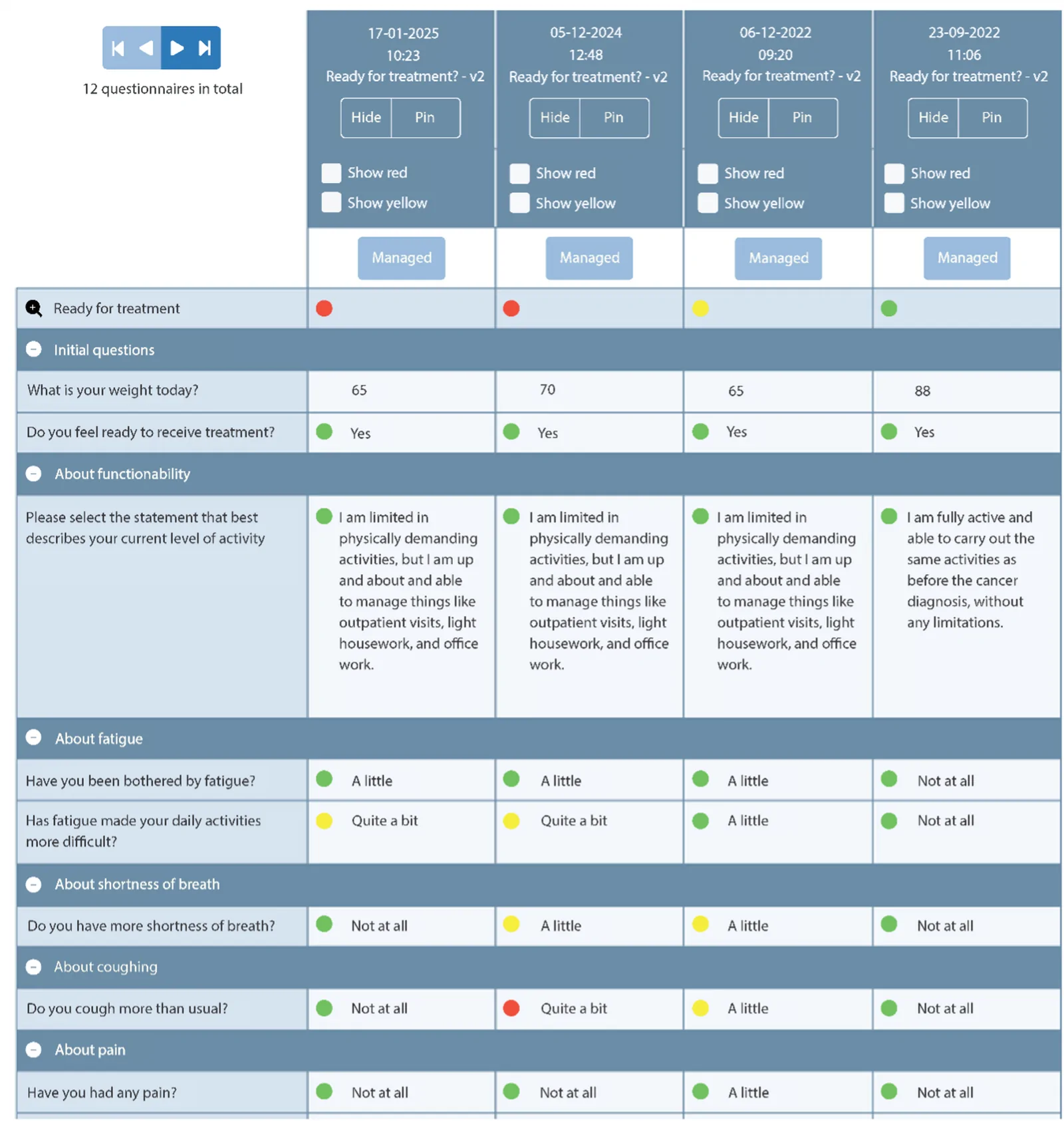
Clinician-view of the ePRO system “Ready for treatment” (Danish: Klar til behandling), showing the first six questionnaire sections and their corresponding items. In this example, the clinician can view the four most recent responses from a total of 12 questionnaire submissions. At the top of the interface, responses can be hidden or pinned, and the display can be filtered to show only yellow or red items. Clinicians can also indicate whether a response has been managed. Below the ‘Managed’ button, the overall summary indicator is shown as green, yellow, or red. Questionnaire items and patient responses are displayed with green, yellow, or red colour coding according to the algorithm (see Supplementary Table 1)

Responses are colour-coded at item level and processed by an algorithm into a green, yellow, or red summary indicator. Green indicates readiness to proceed with treatment as planned without further evaluation, whereas yellow and red require professional assessment to determine whether clinical contact is needed before providing treatment. Questionnaire items, response categories, and the colour coding assigned to each response category are shown in Supplementary Table 1. Clinicians review this output alongside blood test results and medical records to decide whether treatment can proceed. Patients deemed ready receive a message in Mit Sygehus confirming readiness to proceed with treatment. If no questionnaire is completed, or if responses are ambiguous or indicate potential concerns, a nurse contacts the patient by telephone the day before treatment, using the response (if available) to guide the conversation. Figure 1 presents the clinician-facing view of the system.

### Data sources and collection

#### System- and registry data

Questionnaire and clinical data were obtained from 1 December 2020, when the ePRO system was implemented, through 31 March 2024.

Questionnaire data were extracted from ePRO-Syd, the regional IT infrastructure used to manage patient-reported data collected in “Mit Sygehus”. These included metadata such as timestamps (e.g. time of distribution and completion) as well as responses and results of the colour-coded algorithm from completed questionnaires.

Clinical data were extracted from the electronic health record (EHR) system and included information on the number and type of systemic treatments administered. Treatments were identified using the Danish Health Care Classification System (Sundhedsvæsenets Klassifikationssystem, SKS), a standardised national coding system for procedures and diagnostics [22]. All procedures coded as BWHA* and BOHJ*, which relate to systemic cancer therapies and immunomodulatory treatments, registered within the lung cancer unit during the study period were included in the analysis. The procedure codes were selected in consultation with medical experts.

To assess whether a questionnaire had been completed before a treatment session, ePRO-Syd data were linked with EHR records via the unique Danish personal identification number (CPR) [23]. A questionnaire was defined as completed for a given treatment if submitted within seven days prior to the recorded procedure. This timeframe reflects clinical practice, where patients are routinely asked to complete the questionnaire within the days leading up to treatment, capturing relevant symptom information while excluding unrelated entries. The linkage enabled estimation of the overall adoption rate of the ePRO system.

#### Observational fieldwork

Structured field observations were conducted over three consecutive days (26–28 August 2024) at the Oncology Department by two evaluation consultants from the Center for Innovative Medical Technology (CIMT). The purpose was to examine how clinical staff used and experienced the ePRO system in daily practice, focusing on workflow integration and time use.

Observers shadowed one nurse per day, each responsible for patients with lung cancer, enabling observations of concrete actions and to capture what was not explicitly stated [24]. Observations also captured interactions with other staff in the treatment rooms, where both direct care and administrative tasks (e.g., reviewing ePRO data) occurred. Informal conversations with staff were used to capture spontaneous reflections on the ePRO system’s perceived value [25].

An observation guide based on the study objectives was developed to ensure consistent focus. Field notes were taken throughout and served as the primary data source for thematic analysis and for time-use estimation in the evaluation of system-level aspects [26]. The observation guide can be obtained from the corresponding author on request.

#### Patient interviews

Semi-structured telephone interviews were conducted to explore patients’ experiences with and perceptions of the ePRO system, as well as reasons for non-use. Patients were purposively recruited during outpatient visits to ensure variation in age, gender, and ePRO use. Recruitment and data collection occurred from April to June 2024.

An evaluation consultant from CIMT conducted all interviews. A study-specific interview guide was developed based on the purpose of the ePRO system, the evaluation aims, and initial observations in the clinic. Topics included usability, perceived relevance, trust in the system, and barriers to use. The guide was reviewed by clinical and evaluation experts for clarity and relevance prior to use. Interviews were audio-recorded, transcribed verbatim, anonymised, and thematically analysed. The interview guide can be obtained from the corresponding author on request.

Rather than applying a formal data saturation criterion, sample size was guided by pragmatic resource constraints and the aims of this evaluation. Furthermore, the final interviews provided no substantively new insights, suggesting thematic sufficiency.

### Analysis

Quantitative data were analysed descriptively to determine ePRO completion rates, and the proportion of treatments preceded by a completed questionnaire in the period from 1 December 2020 to 31 March 2024. Adoption rate was calculated as the percentage of treatment procedures with a completed questionnaire within the seven-day pre-treatment window. Adaption rates were calculated cross-sectionally and reported on a yearly basis (2020–2024). All quantitative analyses were descriptive and conducted at the level of treatments and completed ePRO questionnaires; we did not perform regression analyses or other inferential statistics.

Qualitative data (interviews and observations) were analysed thematically inspired Braun and Clarke’s six-phase approach [26]. Codes were developed inductively and grouped into overarching themes reflecting implementation processes, workflow adaptations, and perceived benefits or challenges.

Findings from quantitative and qualitative analyses were triangulated within the RE-AIM framework. System and registry data informed Reach/Adoption, observational data captured Implementation, and interview data contributed to inform Perceived Effectiveness. Changes in time use were estimated using modelled workflow scenarios. These scenarios combined registry data on the proportion of treatments with a completed questionnaire with data from field observations and staff interviews. Best-case and worst-case sensitivity analyses were performed by varying key assumptions to illustrate the potential range of estimated time use.

All quantitative data were analysed using Stata version 17.0. Qualitative synthesis and thematic coding were performed in Microsoft Word and Excel.

### Ethics

This study was conducted as part of a quality improvement initiative initiated by the Region of Southern Denmark and is registered in the region’s internal project registry (project no. 24/15914). The project involved no intervention beyond routine clinical practice. According to regional procedures for quality improvement initiatives, no further approval was required.

Interview participants received verbal and written information about the project and provided informed consent prior to participation. Observational fieldwork involved only clinical staff, who were informed in advance.

## Results

The findings are organised according to the RE-AIM framework and presented across three sections corresponding to the dimensions examined in this study:

1. Reach and adoption of the ePRO system, describing patient-level uptake and treatment-level questionnaire completion prior to systemic therapy (RE-AIM: Reach/Adoption);
2. Staff workflow and time use, examining how the ePRO system was implemented in daily clinical practice and its influence on work routines (RE-AIM: Implementation);
3. Patient and staff experiences, exploring perceived benefits, challenges, and overall impact on care delivery (RE-AIM: Perceived Effectiveness).

### Adoption rate and respondent characteristics

Table 1 presents respondent characteristics, questionnaire response distributions, and adoption rates of the ePRO system. In total, 1,014 unique patients received treatment between 1 December 2020 and 31 March 2024. Of these, 704 patients completed at least one ePRO questionnaire before treatment. Slightly more respondents were women (53%), and the mean age was 67 years (range: 39–89). Across all participants, 6,134 questionnaires were completed, with a median of five per patient (range: 1–38).

In terms of summary indicator colours, 8% of questionnaire responses were classified as “green,“, 43% were “yellow,” and 49% were “red”. These distributions remained stable throughout the study period.

Treatment-level adoption, measured as and proportion of treatments where an ePRO questionnaire were completed before treatment, increased markedly over time, rising from 17.7% in 2020 to 76.3% in 2024.

**Table 1:** Respondent characteristics and adoption rate

Variable	*N*	(%)^a^
Unique patients^b^	1,014	
Unique respondents^b^	704	
*Gender*		
Men	329	(46.7)
Women	375	(53.3)
Mean age (SD)	67.4	(8.6)
*Distribution of responses by summary indicator colour*		
Green	471	(7.7)
Yellow	2,656	(43.3)
Red	3,007	(49.0)
Total	6,134	(100)
Number of treatments with a completed ePRO questionnaire		Adaption level^d^
2020^c^	29	(17.7)
2021	1,139	(29.3)
2022	1,790	(52.1)
2023	2,430	(67.5)
2024^c^	592	(76.3)

a Parentheses are percentages unless else stated; b Number of unique patients and unique respondents of the ePRO questionnaire in the period December 2020 through March 2024; c 2020 only had data for December. 2024 only had data for January through March; d The adaption level is the proportion of treatments where an ePRO questionnaire were completed before treatment; Abbreviations: N, Number; SD, Standard deviation; ePRO, Electronic patient-reported outcomes.

### Staff workflow and time use

Implementation of the ePRO system coincided with changes in how nursing staff prepared patients for treatment. Before implementation, all patients were contacted by telephone ahead of each treatment session to assess symptoms, side effects, and overall readiness. Following implementation, the pre-treatment review process became partly digital, combining ePRO questionnaire responses with recent blood test results and electronic health records.

Nurses typically reviewed ePRO responses each morning when preparing treatment lists for the following day. Each patient’s readiness was assessed by combining the color-coded summary indicator with clinical information such as recent blood test results and prior treatment notes. When the summary was not green, nurses opened the full questionnaire and used the item-level colour flags to identify which symptoms required attention. The colours provided a visual overview, allowing staff to focus on areas of potential concern. Nurses also compared current responses with previous entries; for example, if a symptom had been yellow at the last treatment and remained unchanged, it was often not considered clinically significant.

In practice, this translated into a selective approach to follow-up: patients with green responses were rarely contacted, while only about half of those with yellow or red summaries required a phone call. This reflected an integrated decision-making process in which ePRO data were reviewed alongside blood test results and prior responses to determine whether further contact was necessary. Thus, in practice the algorithm-based summary indicator was used pragmatically to guide decision making rather than a stand-alone indicator.

### Estimation of time use

**Fig. 2:**
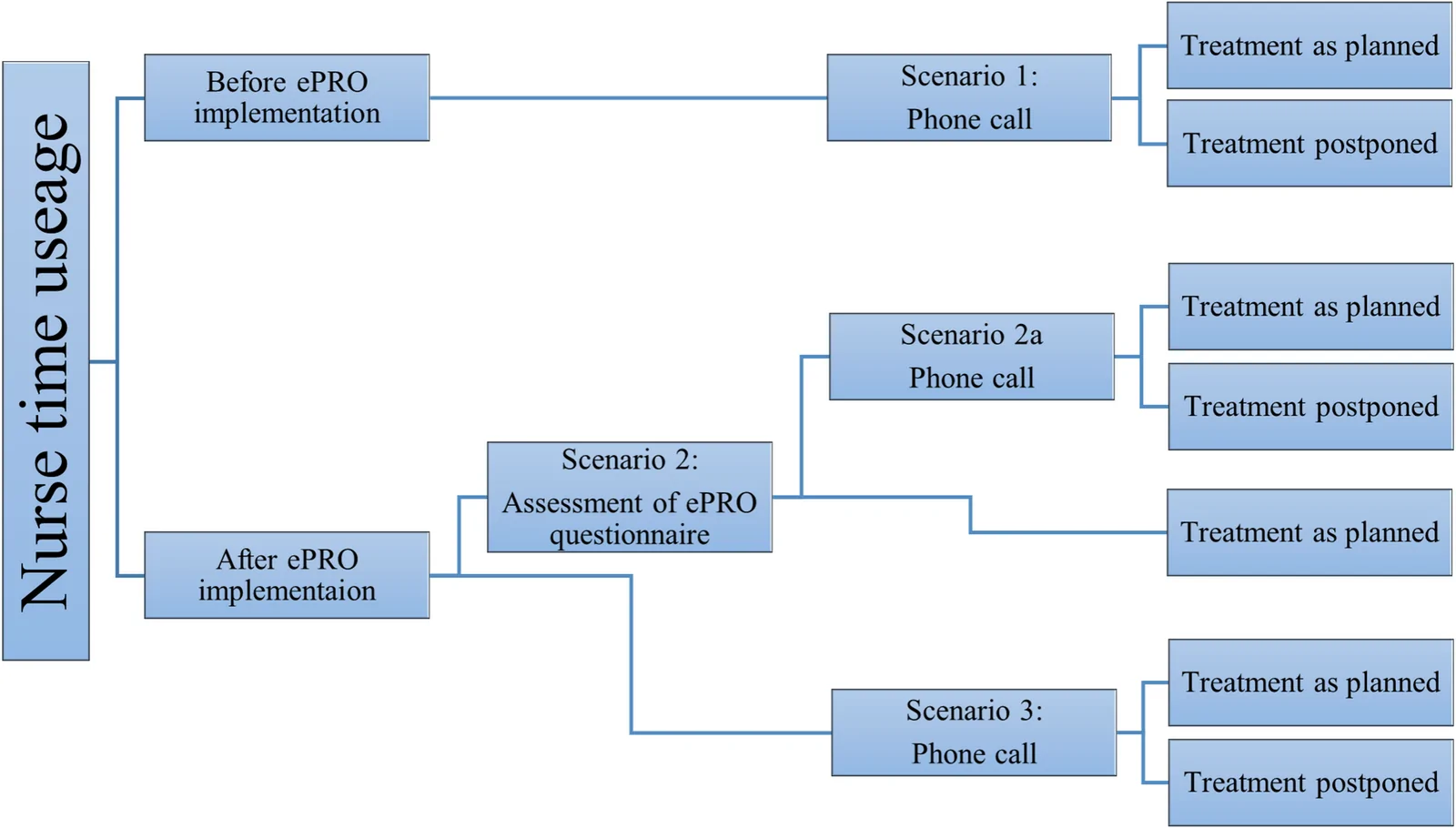
Workflow underlying the time-use estimations before and after implementation of the ePRO system. Before implementation, all patients underwent telephone-based pre-treatment assessment (Scenario 1). After implementation, patients either had a completed ePRO questionnaire reviewed by a nurse (Scenario 2) or were contacted by telephone if no questionnaire had been completed (Scenario 3). A subset of patients with a completed questionnaire also required follow-up by telephone after review (Scenario 2a). Each pathway ended in either treatment as planned or treatment postponed. Diagram was created in Microsoft Word. Abbreviations: ePRO: Electronic patient-reported outcomes

The analysis focused on nurses’ tasks related to treatment preparation, including patient contact, assessment of treatment readiness based on ePRO questionnaires, blood test results, and prior medical history, as well as time spent ordering and preparing medication. Figure 2 shows the workflow before and after implementation. Before implementation, all patients were contacted by telephone prior to treatment (Scenario 1). After implementation, patients were asked to complete the ePRO questionnaire before treatment. Because the completion rate was below 100%, nurses either assessed a completed questionnaire (Scenario 2) or contacted the patient by telephone as before implementation if no questionnaire had been completed (Scenario 3).

Table 2 shows the assumptions, values, and data sources used in the time-use analysis. The final two rows present the estimated time use per treatment and the change relative to pre-implementation practice. Average time use was estimated by multiplying the time required in each scenario by the proportion of patients in that scenario. Before implementation, nurses spent approximately 15 min per treatment. After implementation, estimated time use was around 9 min per treatment, corresponding to an estimated reduction of around 6 min per treatment based on the modelled scenarios.

**Table 2:** Assumptions and values used in the modelled time-use estimations. Values in the base-case column were derived from registry data, field observations, and staff interviews. The best-case and worst-case scenarios represent illustrative sensitivity analyses in which key assumptions were varied in a more favourable or less favourable direction to show the resulting range of estimated time use

Assumption	Base case	Best case	Worst case	Source	Comment
Scenario 1: Proportion of patients with a phone call before implementation	100%	100%	100%	Assumption	
Scenario 2: Proportion of treatments with a completed questionnaire	76%	86%	66%	Registry data	Reflecting questionnaire completion, calculated as the proportion of treatments with a completed questionnaire within a seven-day window
Scenario 2a: Proportion requiring a phone call after review of the ePRO questionnaire	46%	30%	70%	Fieldwork observations, informal staff interviews	
Scenario 1 + 3: Average duration of phone call to patients without a completed ePRO questionnaire	15 min	15 min	15 min	Fieldwork observations, informal staff interviews	This estimate includes cases in which patients did not answer the phone and repeated call attempts were required
Scenario 2a: Average duration of phone call to patients with a completed ePRO questionnaire	10 min	5 min	15 min	Fieldwork observations, informal staff interviews	These calls were generally shorter because the nurse could use the questionnaire responses to guide the conversation
Scenario 2: Average time to assess completed ePRO questionnaire	3 min	2 min	5 min	Fieldwork observations, informal staff interviews	
**Estimated time use per treatment**	9.4 min	5.1 min	15.3 min		Estimated as the weighted average of time use across scenarios, based on the proportion of patients in each scenario
Change since before implementation of ePRO	-5.6 min	-9.9 min	0.3 min		Time use before implementation of ePRO was assumed to be 15 min per treatment, reflecting time spent on phone calls.

N, Number; SD, Standard deviation; ePRO, Electronic patient-reported outcomes

In sensitivity analyses, values were varied to represent best-case and worst-case scenarios (Table 2). In the best-case scenario, time use after implementation was approximately 10 min shorter per treatment. In the worst-case scenario, time use increased by less than half a minute per treatment.

### Patient and staff experiences

A total of 14 patients were recruited for the interview study, of whom 13 participated, including nine ePRO users and four non-users. Participants ranged in age from 64 to 79 years and included six women and seven men (see Supplementary Table 2).

Across interviews, patients reported high overall satisfaction with care and strong trust in clinical staff, irrespective of ePRO use. However, experiences of pre-treatment assessment differed between users and non-users. Among users, the purpose of the PRO-based screening was generally clear, and the system was perceived as meaningful and a natural part of routine care. Most users described the system as accessible and user-friendly, although some required initial assistance from relatives or staff before managing independently. Patients reported that their symptom reporting was taken seriously and contributed to staff assessments of treatment readiness and provided reassurance that adverse effects would be detected and addressed when relevant.

*“There was one time when I had some stomach problems*,* which I reported*,* and I was called in for a consultation right away. I thought that was appropriate*,* because it meant they identified it immediately - ‘there’s something we need to talk about’[…]. It also showed that my responses were read and followed up promptly.”* (Woman, 68 years).

Users emphasized the importance of honest reporting, noting that treatment delay or adjustment – when indicated – was perceived as being in their own best interest. Most patients reported only mild symptoms or few side effects at the time of the interviews. Completing the questionnaire was by some patients viewed as a convenient alternative to routine telephone contact.

*“Yes*,* I think it is fine [to complete questionnaires as part of the treatment]. And then you do not have to sit and wait for them to call from the clinic.”* (Woman, 79 years).

A subset of patients described increased awareness of their own health status and potential side effects when using the system.

In contrast, non-users described less clarity regarding procedures for assessing treatment readiness, e.g. uncertainty about whether routine telephone contact was conducted before every treatment. Reasons for non-adoption included limited reading and writing skills, restricted digital competencies, and possibly lack of use of the secure digital postbox.

From the staff perspective, the ePRO system provided an overview of reported symptoms and side effects, particularly through item-level colour-coding and the possibility to compare responses over time. This was perceived to support preparedness for treatment decisions and patient dialogue, enabling more focused assessments. In addition, staff described increased flexibility in work routines following implementation, as ePRO responses could be reviewed intermittently during the day rather than requiring fixed telephone schedules. When follow-up contact was required, the ePRO responses were used to guide and structure conversations, allowing attention to be directed towards symptoms of potential clinical relevance rather than conducting comprehensive symptom screening.

Both patients and staff also described limitations of the ePRO system and process. Some patients found that predefined response categories did not always capture the complexity or cause of symptoms, e.g. when symptoms were perceived as unrelated to their treatment.

*“I think*,* for example*,* that a question about how I assess my overall condition would have been very relevant. But that question does not exist […] I do write that I have been coughing a lot*,* but then I may add in the comment field that I have been coughing because I had a bad cold and could hardly speak. I think that is a very relevant additional piece of information*,* so that they understand that the cough is probably not related to my disease*,* but to the fact that I had a bad cold.”* (Male, 76 years).

A minority expressed scepticism or a preference for verbal interaction, and one patient perceived limited impact of ePRO responses on treatment decisions, viewing blood tests and imaging as more decisive. Staff similarly emphasised that ePRO data were interpreted alongside blood test results, prior knowledge of the patient, and clinical judgment, and were not used in isolation when determining treatment readiness.

Some patients identified areas where the ePRO system could further enhance its clinical value, including opportunities for patients to elaborate on responses through additional comment fields (e.g. when symptom gradation felt difficult) and inclusion of additional symptom domains (e.g. dry mucous membranes, nausea and blood pressure).

Overall, patient and staff experiences suggest that the ePRO system was perceived to support patient involvement, transparency, preparedness, and focus in treatment readiness assessments.

## Discussion

The findings suggest that an ePRO-based system for assessing treatment readiness can be integrated into routine oncology workflows in a real-world clinical setting for patients receiving systemic therapy for lung cancer. Unlike many previously studied ePRO interventions in oncology that primarily focus on symptom monitoring during treatment or follow-up, the present implementation used patient-reported data to support pre-treatment readiness assessments, effectively replacing routine telephone-based evaluations prior to systemic therapy. From a RE-AIM perspective, the findings indicate substantial reach and adoption among eligible patients, successful integration into clinical workflows (implementation), and positive experiences among both patients and staff (perceived effectiveness).

A central finding of the present study is that ePRO data were not used as a stand-alone triage mechanism but rather as one input among several in clinical decision-making. Nurses combined questionnaire responses with blood test results, prior patient history, and clinical judgement when assessing treatment readiness. This observation is consistent with previous research suggesting that ePRO data are most useful when integrated into existing clinical assessment processes rather than replacing them entirely [27, 28]. In this way, the ePRO system functioned primarily as structured decision support rather than an automated decision-making tool. Consistent with prior research, nurses appeared to play a central role in reviewing and acting upon patient-reported data, reflecting the key role of nursing staff in ePRO-based models of routine cancer care [9]. Concerns that ePRO-based systems might oversimplify clinical decision-making have been raised in earlier studies, particularly when algorithm-generated alerts are interpreted without contextual clinical information [29]. The present findings suggest that clinicians instead use ePRO data pragmatically as part of a broader clinical assessment.

Integration into existing workflows appears to be a critical factor underlying the observed uptake [9]. Previous implementation studies have highlighted poor compatibility with clinical workflows as one of the most frequently reported barriers to ePRO adoption [30, 31]. In the present setting, the ePRO system replaced an existing process - routine pre-treatment telephone calls - rather than introducing an entirely new clinical task. Within the RE-AIM framework, this finding primarily reflects successful implementation, where alignment with established clinical routines may facilitate sustained integration of the intervention in daily practice. Observations further suggested that the system introduced greater flexibility into staff workflows, as questionnaire responses could be reviewed asynchronously during the day rather than within fixed telephone schedules. The successful integration observed in the present study should be interpreted in light of the local organisational context, including existing digital infrastructure and established clinical routines. Previous research has shown that such contextual factors may strongly influence the implementation and long-term sustainability of ePRO systems in routine cancer care [9, 32].

The implementation also had implications at the organisational level. Time-use analyses suggested that the ePRO-based workflow potentially reduced the time required for treatment preparation by approximately six minutes per treatment in the base-case scenario, although sensitivity analyses indicated that this estimate may vary depending on underlying assumptions. While economic consequences were not formally assessed in this study, previous research has highlighted the potential for ePRO-based systems to improve resource utilisation by enabling more targeted patient contact and reducing unnecessary clinical interactions [27, 33]. The present findings indicate that such efficiencies may also arise when ePRO data are used to support treatment readiness assessments in routine practice. Importantly, the system did not eliminate clinical contact when needed; rather, it allowed staff to focus attention on patients with potential symptoms or concerns.

Patient interviews further suggested that most users perceived the ePRO system as meaningful and acceptable. Participants described the questionnaires as easy to use and valued that their responses were reviewed and acted upon when needed. Similar findings have been reported in previous qualitative studies where patients experienced ePRO-based monitoring as enhancing communication and reassurance [28, 34]. From a RE-AIM perspective, these qualitative findings contribute to understanding the perceived effectiveness of the intervention among both patients and clinical staff. At the same time, the interviews highlighted barriers among a subset of patients, particularly related to digital literacy and reading or writing skills. Such barriers are widely recognised in the ePRO implementation literature and underscore the need for alternative pathways for patients unable to use digital reporting systems [9, 29, 35].

### Strengths and limitations

This study has several strengths. First, it provides real-world evidence on the implementation and routine use of an ePRO system in a clinical oncology setting over an extended period. Much of the existing literature on ePRO implementation is based on pilot studies or controlled research settings, whereas the present study reflects everyday clinical practice. Second, the mixed-methods design enabled triangulation of data from system records, observational fieldwork, and patient interviews, providing complementary perspectives on adoption, workflow integration, and user experiences. Finally, several aspects of the study, including qualitative interviews, underscored an effective implementation of the intervention. A patient compliance reaching 76% was consistent with several Danish ePRO intervention studies including patient with various cancer diagnoses (55–94%) [7, 36–40].

Several limitations should be considered when interpreting the findings. First, the study did not include a control group, which limits the ability to draw causal conclusions regarding the impact of the ePRO system on clinical workflows and time use. Observed changes may partly reflect other developments in clinical practice rather than the intervention itself. Second, the analysis compares data across different time periods, during which other contextual factors - such as changes in treatment protocols, staffing patterns, or organisational priorities - may have influenced the results. The COVID-19 pandemic may also have affected both clinical operations and patient behaviour during the study period.

Third, the study was conducted at a single oncology department and focused exclusively on patients receiving systemic therapy for lung cancer. Furthermore, the implementation took place within a tax-funded public healthcare system characterised by high national digital maturity. As organisational structures, digital infrastructure, and patient populations may vary across settings, the generalisability of the findings to other clinical contexts may be limited. Fourth, estimates of time use were partly informed by observational fieldwork conducted over a relatively short period and involving a limited number of staff members, rather than a formal, empirical time-and-motion study. Therefore, the reported time differences should be interpreted as modelled estimates rather than definitively measured outcomes. Although the observations were structured, they may not fully capture variation in daily workflows.

Fifth, the color-coded algorithm used to indicate treatment readiness was developed based on clinical consensus and has not undergone formal validation against a diagnostic gold standard. While clinical risk is mitigated by the fact that staff used the algorithm strictly as a decision-support tool rather than an automated diagnostic mechanism, future research should formally evaluate the algorithm’s sensitivity and specificity.

Finally, the qualitative interview study included a modest sample of patients, and thematic saturation was not formally assessed. Consequently, it is possible that additional interviews could have revealed further, less common nuances regarding user experiences or implementation barriers. The interviewed patients were generally relatively stable, and none reported side effects that substantially affected treatment. As a result, the qualitative findings may overestimate the general acceptability of the system, as they do not fully capture the perspectives of patients with more severe symptoms, treatment-related complications, greater uncertainty about treatment readiness, or lower digital literacy, who might experience the ePRO platform differently.

### Clinical implications and future research

The findings highlight that patient-reported data may serve purposes beyond symptom monitoring by supporting routine clinical workflows such as pre-treatment readiness assessment. In this study, ePRO data functioned as structured input to clinical decision-making, allowing staff to prioritise contact with patients who required additional evaluation while reducing reliance on routine telephone-based assessments. From an organisational perspective, integrating ePRO systems into treatment preparation may therefore enable more targeted use of clinical resources and greater flexibility in staff workflows. However, realising these benefits depends heavily on local digital infrastructure, baseline workflow efficiency, and sustained clinician buy-in. If staff do not actively review and act upon the data, the system risks becoming a parallel administrative task rather than an integrated clinical tool. Future research should examine the broader organisational and economic consequences of such workflow-oriented applications of ePRO systems and explore strategies to ensure equitable access for patients with limited digital competencies or literacy.

## Conclusion

This study indicates that an ePRO-based system can be integrated into routine care for patients receiving systemic therapy for lung cancer and may support treatment readiness assessments in clinical practice. In this setting, patient-reported symptom data were used as part of broader clinical decision-making and appeared to facilitate more targeted pre-treatment contact and greater flexibility in workflow. The patients generally perceived the system as meaningful and acceptable, although barriers related to digital literacy were identified. These findings suggest that ePRO systems may have potential to support routine treatment preparation in oncology, although their value is likely to depend on local context and on maintaining alternative pathways for patients unable to use digital reporting tools.

## Supplementary Information

Below is the link to the electronic supplementary material.

Supplementary Material 1

## Data Availability

The data generated and analysed during the current study are not publicly available.
