# Supplementary material for: Implementation of an ePRO-based system supporting treatment readiness assessments in lung cancer care: a mixed-methods study
Source: Qual Life Res. 2026 Aug 12;35(9):262. doi: 10.1007/s11136-026-04363-0 (PMC13469498; doi:10.1007/s11136-026-04363-0)
Supplement: Supplementary file 1 [file 11136_2026_4363_MOESM1_ESM.docx]

**Supplementary Table 1.** Questionnaire items, response categories, and the colour coding assigned to each response category**.**

| **Section** | **Question** | **Type** | **Response options and colour** | **Included in summary indicator** |
| --- | --- | --- | --- | --- |
| **Initial questions** (2) | 1. What is your weight today? Enter the number of kilograms | Number |  | No |
|  | 2. Do you feel ready to receive treatment? | Single choice | 1) Yes | Yes |
|  |  |  | 2) No |  |
| **About your functional ability** (1) | 3. Please select the statement that best describes your current level of activity | Single choice | 1) I am fully active and able to carry out the same activities as before the cancer diagnosis, without any limitations | Yes |
|  |  |  | 2) I am limited in physically demanding activities, but I am up and about and able to manage things like outpatient visits, light housework, and office work |  |
|  |  |  | 3) I am up and about and able to care for myself, but I am not able to do any work at all. I am out of bed and active for more than half of my waking hours. |  |
|  |  |  | 4) I am able to carry out the necessary self-care. I need to rest in bed or chair for more than half of my waking hours. |  |
|  |  |  | 5) I need round-the-clock care. I am unable to manage anything on my own. |  |
| **About fatigue** (2) (*Since your most recent treatment …*) | 4. Have you been bothered by fatigue? | Single choice | 1) Not at all | Yes |
|  |  |  | 2) A little |  |
|  |  |  | 3) Quite a bit |  |
|  |  |  | 4) Very much |  |
|  | 5. Has fatigue made your daily activities more difficult? | Single choice | 1) Not at all | Yes |
|  |  |  | 2) A little |  |
|  |  |  | 3) Quite a bit |  |
|  |  |  | 4) Very much |  |
| **About shortness of breath** (1) | 6. Do you experience more shortness of breath than usual? | Single choice | 1) Not at all | Yes |
|  |  |  | 2) A little |  |
|  |  |  | 3) Quite a bit |  |
|  |  |  | 4) Very much |  |
| **About cough** (1) | 7. Do you cough more than usual? | Single choice | 1) Not at all | Yes |
|  |  |  | 2) A little |  |
|  |  |  | 3) Quite a bit |  |
|  |  |  | 4) Very much |  |
| **About pain** (3) (*Since your most recent treatment…)* | 8. Have you had any pain? | Single choice | 1) Not at all | Yes |
|  |  |  | 2) A little |  |
|  |  |  | 3) Quite a bit |  |
|  |  |  | 4) Very much |  |
|  | *If > 1 in question 8:*  9. Please indicate where: | Text | Yellow, if completed | No |
|  | *If > 1 in question 8:*  10. Have the pain worsened? | Single choice | 1) Not at all | Yes |
|  |  |  | 2) A little |  |
|  |  |  | 3) Quite a bit |  |
|  |  |  | 4) Very much |  |
| **About sensory disturbances** (2) (*Since your most recent treatment…)* | 11. Have you experienced sensory disturbances, such as tingling, pins and needles, or numbness in your hands and/or feet? | Single choice | 1) Not at all | Yes |
|  |  |  | 2) A little |  |
|  |  |  | 3) Quite a bit |  |
|  |  |  | 4) Very much |  |
|  | *If > 1 in question 11:* 12. Have your sensory disturbances gone away now? | Single choice | 1) Yes | Yes |
|  |  |  | 2) No |  |
| **About nausea** (3) (*Since your most recent treatment...)* | 13. Have you had nausea? | Single choice | 1) Not at all | Yes |
|  |  |  | 2) A little |  |
|  |  |  | 3) Quite a bit |  |
|  |  |  | 4) Very much |  |
|  | *If > 1 in question 13:* 14. When did the nausea start? | Single choice | 1) Less than 24 hours after the most recent treatment | Yes |
|  |  |  | 2) 1-5 days after the most recent treatment |  |
|  |  |  | 3) More than 5 days after the most recent treatment |  |
|  | *If > 1 in question 13:* 15. Has the nausea gone away now? | Single choice | 1) Yes | Yes |
|  |  |  | 2) No |  |
| **About vomiting** (2) (*Since your most recent treatment…)* | 16. Have you vomited? | Single choice | 1) Not at all | Yes |
|  |  |  | 2) A little |  |
|  |  |  | 3) Quite a bit |  |
|  |  |  | 4) Very much |  |
|  | *If > 1 in question 16:* 17. Are you still vomiting? | Single choice | 1) Yes | Yes |
|  |  |  | 2) No |  |
| **About hearing** (3) (*Since your most recent treatment…)* | 18. Have you experienced problems with your hearing, such as reduced hearing, ringing, or high-pitched tones? | Single choice | 1) Not at all | Yes |
|  |  |  | 2) A little |  |
|  |  |  | 3) Quite a bit |  |
|  |  |  | 4) Very much |  |
|  | *If > 1 in question 18:*  19. Please describe your symptoms: | Text | Yellow, if completed | Yes |
|  | *If > 1 in question 18:*  20. Have your hearing problems gone away now? | Single choice | 1) Yes | Yes |
|  |  |  | 2) No |  |
| **About diarrhoea** (4) (*Since your most recent treatment…)* | 21. Have you had diarrhoea? | Single choice | 1) Not at all | Yes |
|  |  |  | 2) A little |  |
|  |  |  | 3) Quite a bit |  |
|  |  |  | 4) Very much |  |
|  | *If > 1 in question 21:*  22. For how many days did you have diarrhoea? | Single choice | 1) 1-3 days | Yes |
|  |  |  | 2) 4-7 days |  |
|  |  |  | 3) More than 7 days |  |
|  | *If > 1 in question 21:*  23. How many extra bowel movements/stools did you have per day at its worst? | Single choice | 1) 1-3 extra bowel movements | Yes |
|  |  |  | 2) 4 or more extra bowel movements |  |
|  | *If > 1 in question 21:*  24. Do you still have diarrhoea? | Single choice | 1) Yes | Yes |
|  |  |  | 2) No |  |
| **About constipation** (3) (*Since your most recent treatment…)* | 25. Have you had constipation? | Single choice | 1) Not at all | Yes |
|  |  |  | 2) A little |  |
|  |  |  | 3) Quite a bit |  |
|  |  |  | 4) Very much |  |
|  | *If > 1 in question 25:*  26. For how many days were you constipated? | Single choice | 1) 1-3 days | Yes |
|  |  |  | 2) 4-7 days |  |
|  |  |  | 3) More than 7 days |  |
|  | *If > 1 in question 25:*  27. Are you still constipated? | Single choice | 1) Yes | Yes |
|  |  |  | 2) No |  |
| **About skin problems** (5) (*Since your most recent treatment…)* | 28. Have you had skin problems, such as itching, rash, or dry skin? | Single choice | 1) Not at all | Yes |
|  |  |  | 2) A little |  |
|  |  |  | 3) Quite a bit |  |
|  |  |  | 4) Very much |  |
|  | *If > 1 in question 28:*  29. Please specify symptom(s) | Multiple choice | 1) Rash | No |
|  |  |  | 2) Dry skin |  |
|  |  |  | 3) Itching |  |
|  |  |  | 4) Other |  |
|  | *If = 4 in question 29:* 30. Please describe: | Text |  | No |
|  | *If > 1 in question 28:*  31. You may add a photo of the area where you have skin problems | Add-a-photo  (Optional) |  | No |
|  | *If > 1 in question 28:*  32. Compared with you most recent treatment, have you had… | Single choice | 1) Fewer symptoms | Yes |
|  |  |  | 2) Unchanged symptoms |  |
|  |  |  | 3) More symptoms |  |
| **About oral symptoms** (7) (*Since your most recent treatment…)* | 33. Have you had symptoms in your mouth? | Single choice | 1) Not at all | Yes |
|  |  |  | 2) A little |  |
|  |  |  | 3) Quite a bit |  |
|  |  |  | 4) Very much |  |
|  | *If > 1 in question 33:*  34. Have you had redness in your mouth? | Single choice | 1) Not at all | Yes |
|  |  |  | 2) A little |  |
|  |  |  | 3) Quite a bit |  |
|  |  |  | 4) Very much |  |
|  | *If > 1 in question 33:*  35. Have you had soreness in your mouth? | Single choice | 1) Not at all | Yes |
|  |  |  | 2) A little |  |
|  |  |  | 3) Quite a bit |  |
|  |  |  | 4) Very much |  |
|  | *If > 1 in question 33:*  36. Have you had blisters in your mouth? | Single choice | 1) Not at all | Yes |
|  |  |  | 2) A little |  |
|  |  |  | 3) Quite a bit |  |
|  |  |  | 4) Very much |  |
|  | *If > 1 in question 33:*  37. Have you had white patches/coatings (plaques) in your mouth? | Single choice | 1) Not at all | Yes |
|  |  |  | 2) A little |  |
|  |  |  | 3) Quite a bit |  |
|  |  |  | 4) Very much |  |
|  | *If > 1 in question 33:*  38. Have you had dry mouth? | Single choice | 1) Not at all | Yes |
|  |  |  | 2) A little |  |
|  |  |  | 3) Quite a bit |  |
|  |  |  | 4) Very much |  |
|  | *If > 1 in question 33:*  39. Have you had difficulty eating and/or drinking? | Single choice | 1) Not at all | Yes |
|  |  |  | 2) A little |  |
|  |  |  | 3) Quite a bit |  |
|  |  |  | 4) Very much |  |
| **Final questions** (3) | 40. Have you had a fever since your most recent treatment? | Single choice | 1) Yes | Yes |
|  |  |  | 2) No |  |
|  | 41. Have you been hospitalized since your most recent treatment? | Single choice | 1) Yes | Yes |
|  |  |  | 2) No |  |
|  | 42. Have you had any other side effects, or do you have any comments on your response? | Text  (Optional) | Yellow, if completed | Yes |

**Supplementary Table 2:** Respondent characteristics in the interview study. Abbreviations: n: number; ePRO: Electronic patient-reported outcomes; min: minutes

|  | **Users of ePRO (n = 9)** | **Non-users of ePRO (n = 4)** | **Total (n = 13)** |
| --- | --- | --- | --- |
| Mean age (range) | 72 (64-79) | 70 (68-74) | 71 (64-79) |
| Female, n (%) | 4 (44 %) | 2 (50 %) | 6 (46 %) |
| Mean interview length (range), min | 17 (9-24) | 10.3 (7-14) | 14.9 (7-24) |
| ePRO questionnaires completed (range) | 5.5 (5-13) | - | - |
| Declined participation (reason) | 1 (could not be reached) | 0 | 1 |
